# Bub1 Kinase Targets Sgo1 to Ensure Efficient Chromosome Biorientation in Budding Yeast Mitosis

**DOI:** 10.1371/journal.pgen.0030213

**Published:** 2007-11-30

**Authors:** Josefin Fernius, Kevin G Hardwick

**Affiliations:** Wellcome Trust Centre for Cell Biology, Institute of Cell Biology, University of Edinburgh, United Kingdom; National Institute of Diabetes & Digestive & Kidney Diseases, United States of America

## Abstract

During cell division all chromosomes must be segregated accurately to each daughter cell. Errors in this process give rise to aneuploidy, which leads to birth defects and is implicated in cancer progression. The spindle checkpoint is a surveillance mechanism that ensures high fidelity of chromosome segregation by inhibiting anaphase until all kinetochores have established bipolar attachments to spindle microtubules. Bub1 kinase is a core component of the spindle checkpoint, and cells lacking Bub1 fail to arrest in response to microtubule drugs and precociously segregate their DNA. The mitotic role(s) of Bub1 kinase activity remain elusive, and it is controversial whether this C-terminal domain of Bub1p is required for spindle checkpoint arrest. Here we make a detailed analysis of budding yeast cells lacking the kinase domain (*bub1*Δ*K*). We show that despite being able to arrest in response to microtubule depolymerisation and kinetochore-microtubule attachment defects, *bub1*Δ*K* cells are sensitive to microtubule drugs. This is because *bub1*Δ*K* cells display significant chromosome mis-segregation upon release from nocodazole arrest. *bub1*Δ*K* cells mislocalise Sgo1p, and we demonstrate that both the Bub1 kinase domain and Sgo1p are required for accurate chromosome biorientation after nocodazole treatment. We propose that Bub1 kinase and Sgo1p act together to ensure efficient biorientation of sister chromatids during mitosis.

## Author Summary

Many human diseases, including birth defects and cancer, are associated with aneuploidy. This is where cells have an incorrect number of chromosomes, because of a failure to segregate their genetic material accurately during cell division. Cells employ many control mechanisms to ensure an extremely high fidelity of chromosome segregation. One way that they do this is to hold the replicated copies of their chromosomes (known as sister chromatids) together until they are all attached properly to microtubules of the mitotic spindle. All pairs of sister chromatids must have one sister attached to each of the two spindle poles, a process known as biorientation. Here we demonstrate that the Bub1 kinase domain acts to target Sgo1 to budding yeast centromeres, and that both of these proteins are required for efficient biorientation of chromosomes in yeast mitosis. Bub1 kinase and Sgo1 functions become particularly important during spindle reassembly after antimicrotubule drug treatment. We propose that this is because the mutant cells fail to respond to kinetochores that are not under tension, and that they are unable to correct syntelic attachments where both sister chromatids attach to microtubules from the same spindle pole.

## Introduction

The fidelity of chromosome segregation is dependent upon correct bipolar attachment of sister chromatids to the spindle microtubules (for review see [1]). These attachments are mediated through complex, molecular machines called kinetochores, which assemble at the centromere of each chromosome (for reviews see [2,3]). Accurate chromosome segregation is crucial: any errors lead to aneuploidy, which is characteristic of many diseases and a hallmark of tumour progression [4,5]. Cells have evolved a number of control mechanisms to prevent segregation errors. One of the most important is the spindle checkpoint, which is a surveillance system that tightly regulates the metaphase-to-anaphase transition. It ensures that all kinetochores have established proper bipolar (also known as amphitelic) attachments, where sister kinetochores are attached to microtubules emanating from opposite spindle pole bodies (SPBs), before anaphase is initiated [3,6,7]. The spindle checkpoint consists of a set of conserved proteins (Mad1-3p, Bub1p, Bub3p, and Mps1p [8–10]) that form distinct complexes and localise to unattached kinetochores in a highly ordered manner [11,12]. The downstream target of these proteins is Cdc20p [13,14], an activator of the E3 ubiquitin ligase known as the Anaphase Promoting Complex or Cyclosome (APC/C) (for review see [15]). Securin (Pds1p in budding yeast) and cyclin B are the key APC/C substrates and polyubiquitination of these, and their ensuing proteolytic destruction, is required for anaphase onset and mitotic exit [16–18]. Thus when the spindle checkpoint is active, the APC/C is inhibited, Securin levels remain high, and anaphase onset is delayed.

The spindle checkpoint responds to unattached kinetochores [19,20] and to lack of tension across sister kinetochores that have yet to achieve proper biorientation [21]. These two responses are linked through the Aurora B kinase homologue, Ipl1p (see [22] for review). Ipl1p is not required to activate the spindle checkpoint in the response to unattached kinetochores induced by antimicrotubule drugs, but is required to respond to a lack of tension [23]. Ipl1p recognises and breaks defective or inappropriate kinetochore-microtubule attachments, which lack tension, and thereby creates unattached kinetochores [24]. In addition, Ipl1p-dependent phosphorylation of Mad3p ensures full inhibition of Cdc20-APC/C in cells with reduced cohesion [25].

Other protein kinases (Bub1, BubR1, and Mps1) also play an integral part in the spindle checkpoint (see [6]). Bub1p is a conserved protein kinase, which is essential for spindle checkpoint arrest. Bub1p forms a complex with Bub3p [26], binds Mad1p when the checkpoint is active [27], and is required for localisation of several checkpoint proteins to unattached kinetochores [12,28,29]. It is well established that the N-terminal domains of Bub1p, which include the kinetochore targeting and Bub3p-binding domains, are required for spindle checkpoint arrest [29,30]. However the role of the C-terminal kinase domain remains elusive, although it has been suggested that phosphorylation of Cdc20 by human Bub1 is required to enhance the inhibition of APC/C [31]. Several lines of evidence suggest role(s) for Bub1 in addition to its spindle checkpoint function. Bub1 is required for accurate chromosome segregation in both budding and fission yeasts [29,30] and to maintain ploidy in fission yeast [32]. In addition, there is evidence that Bub1 plays a role in chromosome congression in mammalian cells [28,33].

In this paper we characterise the role of the Bub1p kinase domain in budding yeast mitosis. We show that *bub1*Δ*K* cells die rapidly in the presence of microtubule drugs despite being able to initiate and maintain a spindle checkpoint arrest. This rapid cell death is due to chromosome mis-segregation following release from antimicrotubule drugs. In addition, we demonstrate a role for Bub1 kinase in accurate Sgo1p localisation to mitotic centromeres. Sgo1p is the sole budding yeast member of the Shugoshin/MEI-S332 family (for review see [34]). Members of this family are important protectors of centromeric cohesion, particularly in meiosis [35–38]. However, budding yeast Sgo1p does not regulate cohesion in budding yeast mitosis, and it has been proposed that this protein is a tension sensor at kinetochores [39]. Here we demonstrate that the Bub1 kinase domain and Sgo1p act together to ensure efficient chromosome biorientation.

## Results

Although Bub1 kinase activity has been shown to be required for accurate chromosome segregation in both budding and fission yeasts [29,30], the roles of the Bub1 kinase domain have not been clearly established. Indeed, it remains controversial whether the kinase activity is required for a spindle checkpoint arrest, in either organism. In some budding and fission yeast reports, Bub1 kinase activity was thought to be necessary for checkpoint arrest [26,40], but in others it was not [29,30]. This controversy might partly be due to the use of a “kinase-dead” point mutation (K733R), which has since been shown to destabilise budding yeast Bub1p [30] and could therefore display a phenotype similar to that of a *bub1*Δ. We generated a novel K733M kinase-dead allele, but found that this protein was also unstable (K. G. Hardwick, unpublished data). Therefore, we chose to carry out a detailed analysis of the role of the Bub1 kinase domain by using a truncated Bub1 kinase allele (containing amino acids 1–608) in *Saccharomyces cerevisiae.* This allele lacks the whole kinase domain and has already been shown to express a stable protein [30], and to efficiently bind Bub3p and Mad1p [27]. A similar Bub1 truncation is stable and is able to localise to kinetochores in S. pombe ([40] and V. Vanoosthuyse, personal communication).

### Cells Lacking the Bub1 Kinase Domain Die Rapidly in the Presence of Microtubule Depolymerising Drugs

**Figure 1:**
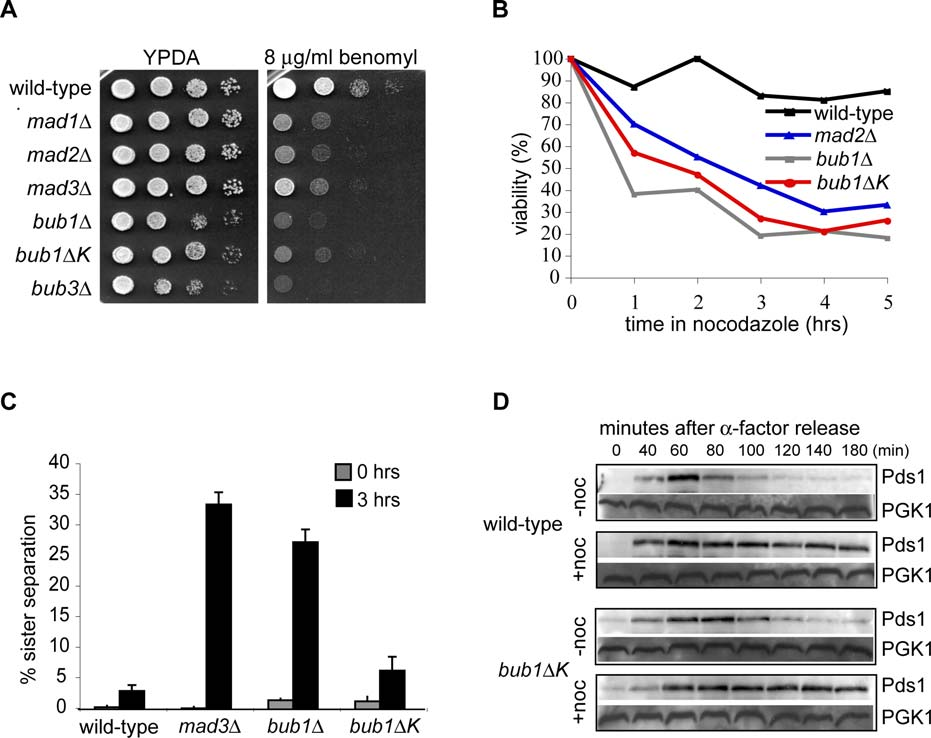
Cells Lacking Bub1 Kinase Domain Show Sensitivity to Microtubule Depolymerising Drugs despite Being Spindle Checkpoint Proficient (A) Wild-type (KH186), *mad1*Δ (MB076), *mad2*Δ (KH141), *mad3*Δ (KH173), *bub1*Δ (KH127), *bub3*Δ (MB003), and *bub1*Δ*K* (JF098) strains were plated out in 10-fold serial dilutions on YPDA media or on YPDA media containing 8 μg/ml benomyl. (B) The indicated strains were grown in YPDA media containing 30 μg/ml nocodazole, and the viability of the cells was measured as percentage of cells able to form colonies on YPDA media lacking microtubule drugs. (C) Wild-type (JF004), *bub1*Δ*K* (JF125), *bub1*Δ (JF140), and *mad3*Δ (EK013) strains containing a GFP-marked chromosome were synchronised in G1 using α-factor, then released into YPDA with 30 μg/ml nocodazole at 23 °C. We tested ability of the cells to maintain a spindle checkpoint arrest by scoring cells that could keep their GFP-marked sister chromatids cohesed for 3 h in nocodazole (i.e., one GFP dot). The percentage of cells with two GFP dots was counted at the release from G1 (grey bars) and after 3 h in nocodazole (black bars) (*n* = 100 cells for each repeat experiment). Error bars indicate standard deviation. (D) Wild-type (JF004) and *bub1*Δ*K* (JF125) strains containing Pds1-18myc were arrested in G1 using α-factor and synchronously released into YPDA media or YPDA media containing 30 μg/ml nocodazole at 23 °C. Samples were taken at indicated times. Levels of Pds1 were monitored by immunoblotting using A14 α-myc antibody and α-PGK1 as a loading control.

Spindle checkpoint mutants are hypersensitive to microtubule destabilising drugs because of their inability to arrest in metaphase in response to unattached kinetochores. The precocious separation of sister chromatids gives rise to unequal segregation of chromosomes and aneuploidy, which is lethal in yeast. On rich media containing low concentrations of the microtubule drug benomyl, *bub1*Δ*K* cells showed an intermediate sensitivity to the drug compared to other spindle checkpoint mutants. For example, it was clear that the *bub1*Δ*K* mutation was not as benomyl sensitive as the complete *bub1*Δ but that it was more sensitive than *mad3Δ* (Figure 1A). Spindle checkpoint mutants die rapidly in liquid cultures containing microtubule drugs [41], so we asked how long *bub1*Δ*K* cells remain viable under such conditions. Cells were grown in liquid media containing 30 μg/ml nocodazole and then plated on rich media lacking microtubule drugs. Viability was scored as the percentage of cells able to form colonies. In contrast to wild-type cells, *bub1*Δ*K* behaved like *bub1*Δ and *mad2*Δ cells and showed rapid death in this viability assay (Figure 1B). After 1 h, 55% of *bub1*Δ*K* cells were already inviable. These data show that cells lacking the Bub1 kinase domain are sensitive to microtubule drugs and that they die rapidly, but that *bub1*Δ*K* is not a complete loss of function (null) allele.

### The Bub1 Kinase Domain Is Dispensable for a Robust Spindle Checkpoint Arrest in Response to Unattached Kinetochores

*bub1*Δ*K* cells could be sensitive to microtubule depolymerising drugs for various reasons. They may be unable to arrest in mitosis, or they may fail to recover properly after spindle checkpoint arrest. To distinguish between these possibilities, we examined cell morphology and the level of sister-chromatid cohesion in *bub1*Δ*K* cells in response to microtubule drugs. First we performed a morphological assay scoring rebudding of cells on plates containing 20 μg/ml and 80 μg/ml benomyl (see Figure S1). Both wild-type and *bub1*Δ*K* cells remained large-budded (which is an indication of mitotic arrest) for up to 6 h on benomyl, compared to the spindle checkpoint mutants, *mad2*Δ and *bub1*Δ, which did not respond to microtubule depolymerisation and rebudded prematurely. This confirms the previous report of a large-budded arrest for *bub1ΔK* cells in nocodazole [30]. To monitor sister-chromatid cohesion directly, we used strains expressing lacI-green fluorescent protein (GFP) and containing a lac-operator array on Chromosome IV, thereby marking this chromosome with GFP. This enabled us to visualize whether chromatids were still cohesed (one GFP dot) or had separated prematurely (two GFP dots) in the presence of antimicrotubule drugs. Cells were synchronized in G1, using the mating pheromone α-factor, and released into media containing nocodazole. *bub1*Δ*K* cells behaved like wild type and efficiently maintained sister-chromatid cohesion for 3 h in cultures containing nocodazole (Figure 1C). As a final test of mitotic arrest, we asked whether Securin (Pds1-myc) was stabilized in the presence of nocodazole in *bub1*Δ*K* cells. This was the case (Figure 1D), confirming that the *bub1*Δ*K* cells efficiently respond to microtubule depolymerisation and initiate and maintain a robust spindle checkpoint arrest.

### The Bub1 Kinase Domain Is Necessary for the Checkpoint Response to Reduced Cohesion

**Figure 2:**
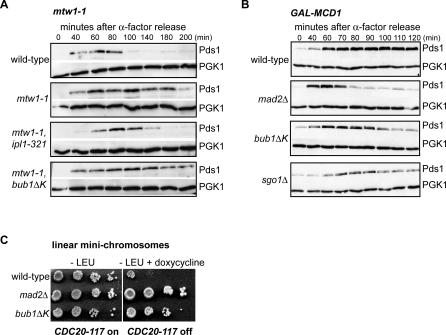
Bub1 Kinase Domain Is Required to Arrest in Response to Reduced Cohesion (A) Wild-type (JF004), *mtw1–1* (SBY1646), *mtw1–1*, *bub1*Δ*K* (JF100), and *mtw1–1*, *ipl1*-321 (SBY1724) strains carrying the temperature-sensitive allele *mtw1–1* and Pds1-18Myc were synchronised in G1 for 2.5 h then incubated at the restrictive temperature 36 °C for 30 min before release. The levels of Pds1 were monitored by immunoblotting with A14 anti-myc antibody and anti-PGK1 as a loading control. (B) Wild-type (VBI545), *bub1*Δ*K* (JF023), *mad2*Δ (VBI560), and *sgo1*Δ (JF224) strains, containing Pds1-myc, were synchronised in G1 with α-factor in media containing 2% galactose and 2% raffinose to maintain Mcd1 expression. Mcd1 was then turned off by incubating them in media containing glucose and α-factor for 3 h. The cells were then released into rich media containing glucose, and samples for immunoblotting were taken at indicated times. Pds1 and PGK1 levels were monitored as previously described. (C) Wild-type (YBS151), *bub1*Δ*K* (JF110), and *mad2*Δ (YBS406) strains, carrying a *LEU2*-marked linear minichromosome (LMC) were plated in 10-fold serial dilutions on −LEU plates and on −LEU plates with 10 μg/ml doxycycline. In this strain doxycycline represses a dominant *CDC20* allele that is insensitive to the spindle checkpoint proteins [13]. Therefore this *CDC20* allele makes cells insensitive to the presence of LMCs. These LMCs are thought to be unable to withstand spindle forces and separate producing kinetochore-microtubule attachments that are no longer under tension. In the presence of doxycycline, wild-type cells grow very poorly because of their checkpoint response to the LMCs, but the *bub1*Δ*K* mutant grows far better, indicating that their ability to respond to attachments lacking tension is impaired.

In the assays described above, we used antimicrotubule drugs to activate the spindle checkpoint pathway that recognizes unattached kinetochores. Complete microtubule depolymerisation is not a very common physiological situation, and we were therefore interested to analyse *bub1*Δ*K* cells in other situations. Ipl1 kinase activates the spindle checkpoint by creating unattached kinetochores in response to mutations in several kinetochore components that were thought to create reduced tension at centromeres [24]. We wondered whether the kinase domain of Bub1 is required to arrest cells containing such defective kinetochores. In contrast to an *ipl1–321, mtw1–1* double mutant, cells containing the *mtw1–1* mutation in combination with *bub1*Δ*K* were able to respond to the kinetochore defect and stabilise Pds1-myc (Figure 2A). Similar results were obtained when using the more severe *ndc80–1* kinetochore mutant in combination with *bub1ΔK* (unpublished data). These results show that Bub1 kinase is not required to “sense” these defective kinetochores, nor to activate Ipl1p in these mutants, nor to respond to the unattached kinetochores that Ipl1p kinase activity creates.

Ipl1p (Aurora kinase), Mad3p phosphorylation, and Sgo1p have all been demonstrated to be necessary for the checkpoint response to reduced cohesion, even though they are not necessary for the response to unattached kinetochores [23,25,39]. To test whether the Bub1 kinase domain is necessary for the response to reduced cohesin, *bub1*Δ*K,GAL-MCD1* cells were synchronised in G1, depleted for cohesin by turning off *MCD1* expression with glucose addition, and then released into the cell cycle. Immunoblotting for Securin levels (Pds1) showed that neither *bub1*Δ*K* nor *sgo1* mutants were able to maintain a metaphase arrest as well as wild-type cells (Figure 2B). They did not degrade Pds1p quite as quickly as the *mad2*Δ control, but we believe that was because they grow more slowly, most likely because of their aneuploidy phenotype. We also analysed the ability of *bub1ΔK* cells to grow in the presence of poorly segregating linear chromosomes. Such chromosomes have been shown to delay mitosis in a checkpoint-dependent manner [42] and were employed in a screen that identified *sgo1* alleles [39]. For the screen, the linear chromosome-induced delay becomes lethal as the strains also contain *CDC28-VF,* a mutation in *CDC28/CDK1* that reduces APC activity. This lethality is rescued by checkpoint mutations, as no mitotic delay is imposed. Figure 2C shows that *bub1ΔK* cells grow far better than wild type in the presence of the short linear chromosomes, indicating that this checkpoint response is also defective. We conclude that whilst the Bub1 kinase domain is not necessary for the response to unattached or defective kinetochores, it is necessary for the budding yeast checkpoint response to a lack of tension at mitotic kinetochores.

The above experiments strongly suggest that the C-terminal kinase domain of Bub1 is not required to initiate or maintain spindle checkpoint arrests induced by unattached or defective kinetochores, but that it is necessary to respond to a lack of tension. The *bub1ΔK* phenotype closely mirrors that of *sgo1Δ*, which is also necessary for the tension response [39] but not the response to *mtw1–1*- or *ndc80–1*-induced kinetochore defects [24]. The *bub1ΔK* and *sgo1Δ* phenotypes differ from that displayed by *ipl1* mutants. Ipl1p is required for the tension response but also for the response to *mtw1–1*- or *ndc80–1*-induced kinetochore defects [24], and Ipl1p is an essential protein [43].

### The Bub1 Kinase Domain Plays a Role in Sgo1 Localisation

**Figure 3:**
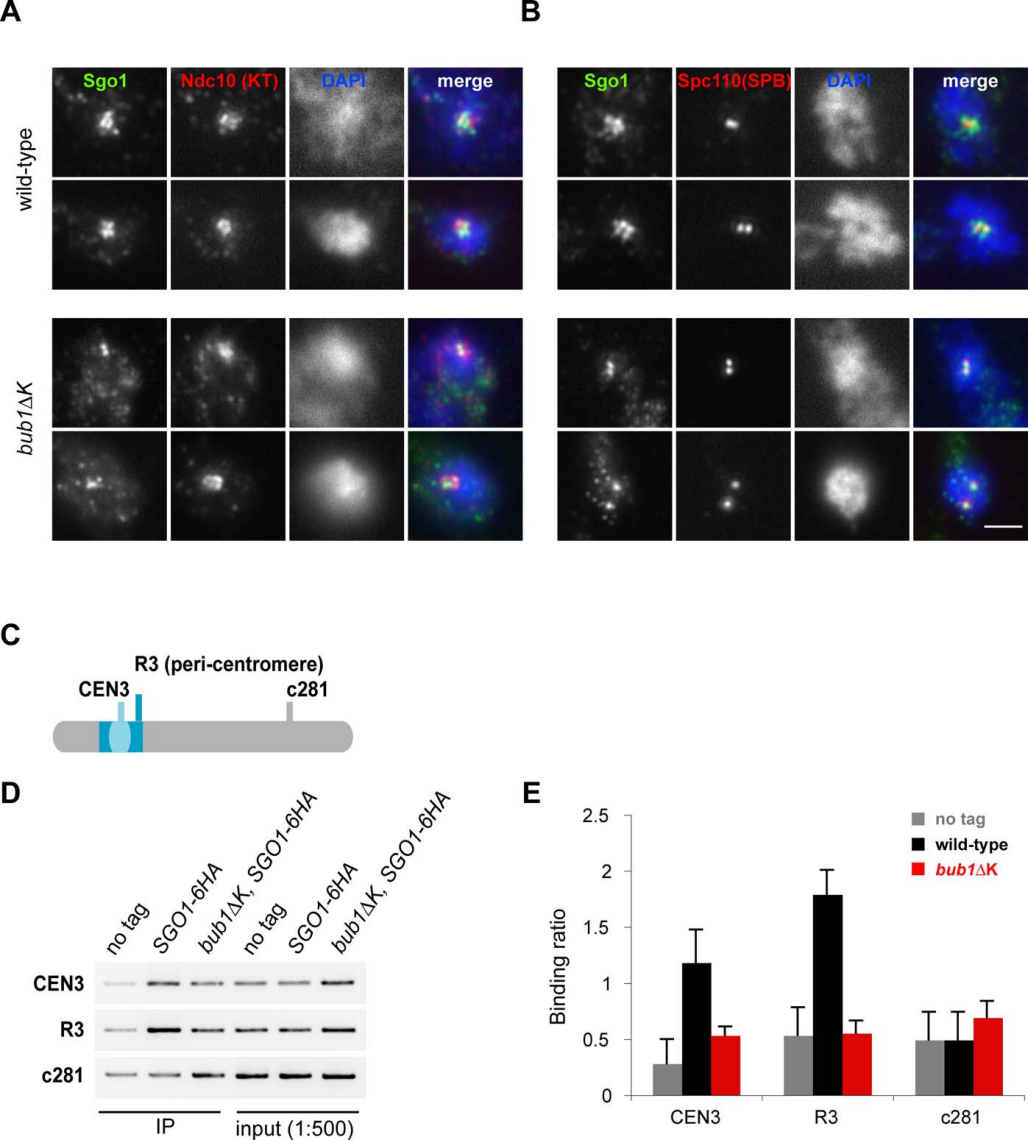
Bub1 Kinase Domain Is Necessary for Accurate Sgo1 Localisation to Kinetochores (A) Wild-type (AMY1110) and *bub1*Δ*K* (JF038) strains containing Sgo1-9Myc and Ndc10-6HA (to mark the kinetochores) were arrested in metaphase with 15 μg/ml nocodazole and 30 μg/ml benomyl at 23 °C for 3 h. Chromosome spreads were performed and stained with α-myc antibody (CM-100), α-HA antibody (HA11), and DAPI to recognize the DNA. Spreads with clear Ndc10-6HA staining were categorized as showing colocalization with Sgo1-9myc only if they contained multiple (>2) overlapping foci. Fifty spreads per strain were analysed. (B) Sgo1-9myc colocalises with the SPB in the *bub1*Δ*K* mutant. Spreads were prepared as in (A) and stained with α-myc antibody, anti-Spc110 antibody to detect SPBs, and DAPI. Scale bar represents 2 μm. (C) Schematic of primers sets for Sgo1-6HA ChIP analysis showing the CEN3, centromeric region; R3, pericentromeric region; and c281, the negative arm region. (D) PCR on ChIPs of “no tag” (KH186), Sgo1-6HA (AMY209), and *bub1*Δ*K,* Sgo1-6HA (JF211) strains arrested with 15 μg/ml nocodazole and 30 μg/ml benomyl at 23 °C for 3 h, showing reduced Sgo1p levels associated with CEN3 and R3 regions in the *bub1*Δ*K* mutant compared to wild type. (E) The graph shows quantification of the ChIP data from the “no tag” (KH186), wild-type (AMY209), and *bub1*Δ*K* (JF211) strains. The “binding ratio” was calculated as a ratio of the ChIP PCR signal to the PCR signal from a 1:500 diluted input fraction. The error bars indicate standard deviation (SD) of the mean from five different PCR reactions from two separate experiments.

Bub1p is important for Sgo1p localisation at centromeres in meiosis in budding and fission yeast [36,37,44] and regulates Sgo1 localisation in mitosis in human cells [45,46]. Because of this and the similarity in the *bub1ΔK* and *sgo1Δ* phenotypes, we tested whether the Bub1 kinase domain has a role in localising Sgo1p to kinetochores in budding yeast mitosis. Unfortunately Sgo1-GFP gives a rather weak signal, so we did this by performing immunofluorescence on fixed chromosome spreads. We used a strain containing Ndc10-6HA to label kinetochores and asked whether Sgo1-9Myc colocalised with Ndc10p in wild type, *bub1*Δ, and *bub1*Δ*K* cells. We analysed unbiased populations of cycling cells (see Figure S2) and cultures that had been arrested in mitosis for 3 h with nocodazole. Whilst wild-type cells showed multiple foci with overlapping localisation for Sgo1p and Ndc10p in many spreads, this was rarely the case for *bub1ΔK* cells (Figure 3A). Categorization revealed such colocalisation of Sgo1p and Ndc10p in only 13% of mutant cells (compared with 2% in wild type). In *bub1ΔK* cells there were often one or at most two bright punctate signals for Sgo1p. To determine whether these could be SPBs, we carried out double label staining for Sgo1-9Myc and the 110-kD component of the SPB. Colocalization was observed in many cells (Figure 3B), suggesting that significant levels of Sgo1p localise to the SPB in the *bub1ΔK* mutant. We cannot rule out the possibility that some of this signal is due to centromeres that remain associated with the SPB in these nocodazole-treated cells. To confirm the decreased association of Sgo1p with kinetochores in *bub1ΔK* cells, we employed chromatin immunoprecipitation (ChIP). We reproducibly observed decreased association of Sgo1p and centromeres, using both centromeric and pericentromeric primer sets, in *bub1ΔK* cells (Figure 3C–3E).

These results confirm that the kinase domain of Bub1p plays an important role in localising Sgo1p to budding yeast centromeres in mitosis. This could explain why the *sgo1*Δ and *bub1*Δ*K* strains display such similar phenotypes.

### *bub1*ΔK Cells Mis-Segregate Chromosomes during Recovery from a Checkpoint Arrest

**Figure 4:**
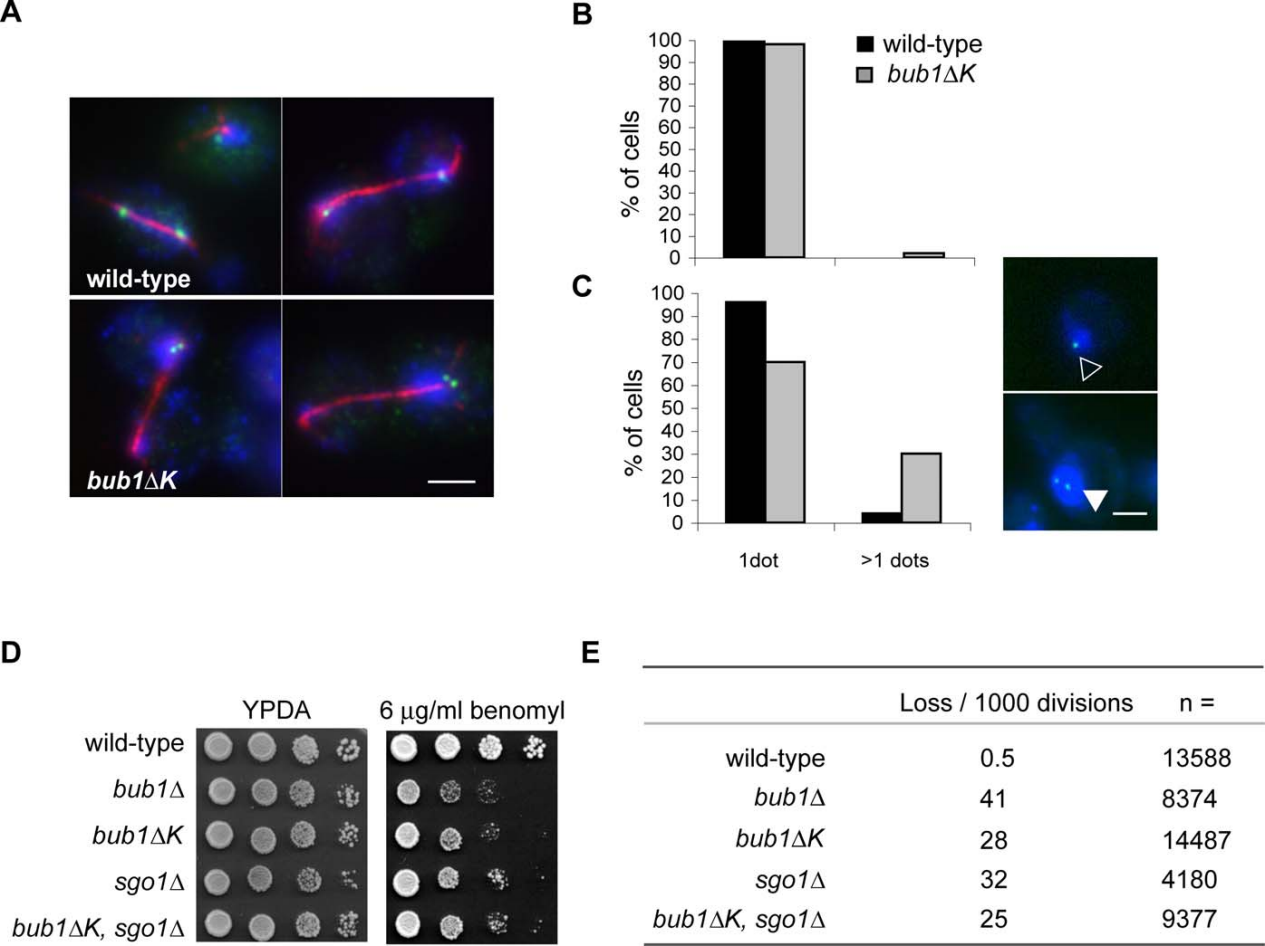
*bub1*ΔK Cells Display High Levels of Chromosome Mis-segregation upon Nocodazole Release (A) Wild-type (JF004) and *bub1*Δ*K* (JF125) cells were released from G1 into media containing 30 μg/ml nocodazole and incubated at 23 °C for 3 h. Cells were subsequently released into anaphase by washing out the nocodazole. Samples were fixed in 3.7% formaldehyde for 1 h, 30 min after release, and stained with α-GFP (GFP-marked chromosome) and α-tubulin (red spindle) antibodies. DNA was stained with DAPI (blue). Percentage of nondisjunction of the GFP-marked chromatid at the first anaphase following nocodazole arrest was 2% in wild-type cells and 33% in *bub1Δ* *K* cells (*n* ≥ 50 anaphase cells). Scale bar represents 3 μm. (B) Wild-type (JF004) and *bub1*Δ*K* (JF125) strains were synchronised in G1 as previously described and cells with one GFP dot (empty triangle) versus two dots (filled triangle) were counted (*n* = 400). Scale bar represents 2 μm. (C) Cells from (B) were then released and incubated in media containing 30 μg/ml nocodazole at 23 °C for 3 h and released into media containing α-factor to score cells in the following G1. The number of cells with one GFP dot versus two dots were scored (*n* = 400). (D) Wild-type (KH186), *bub1*Δ (KH127), *bub1*Δ*K* (JF098), *sgo1*Δ (JF188), and *bub1*Δ, *sgo1*Δ (JF185) strains were plated out in 10-fold serial dilutions on rich media and on rich media containing 8 μg/ml benomyl. (E) Strains carrying the *SUP11* artificial chromosome were grown overnight in CSM-URA media, then diluted back to OD_600_ 0.2 and grown in YPDA media at 30 °C for 3 h. Cells were then plated out on YPD at a density of ∼500 cells per plate. Only colonies that were at least half red were scored for losing the test chromosome at the first division.

The fact that *bub1ΔK* cells are hypersensitive to antimicrotubule drugs, yet able to arrest efficiently, suggested to us that the Bub1 kinase domain could be required for proper recovery from spindle damage. To test this, we first asked whether they have increased chromosome loss following nocodazole arrest. We followed chromosome segregation during the first anaphase after nocodazole release, using the GFP-marked chromosome strain described above. Accurate chromosome segregation should give rise to one GFP spot in each daughter cell. We arrested cells in metaphase for 3 h with nocodazole then released them into anaphase. After 30 min, cells were fixed and stained with α-tubulin antibody to monitor spindle elongation. The results showed 33% mis-segregation of the GFP-marked chromatids in *bub1*ΔK cells (Figure 4A, lower panel) compared to 0.2% in wild type (Figure 4A, upper panel).

We then used the same strain to score a large number of cells for chromosome mis-segregation and compared unchallenged G1 cells with G1 cells that had been released from a nocodazole arrest. Cells were arrested in G1 using α-factor, and the number of cells with one GFP foci (Figure 4C, empty triangle) versus two GFP foci (filled triangle) were counted. As expected, most cells had one GFP dot, representing one copy of Chromosome IV, in both wild-type (0% had two GFP dots, *n* = 400) and *bub1*ΔK cells (2% had two GFP dots, *n* = 400). The small number of *bub1ΔK* cells with two copies of this chromosome (Figure 4B) reflects a background level of aneuploidy, frequently observed in *bub1* mutants. However, when cells were released from G1 into media containing nocodazole for 3 h, then released and trapped in the following G1, there was a marked increase in the number of cells containing two GFP foci in *bub1*Δ*K* (30% had two GFP dots, *n* = 400) compared to wild type (where only 4% had two GFP dots, *n* = 400) (Figure 4C). This confirms that there was a significant defect in segregating this chromosome faithfully during the anaphase following nocodazole release. Because of the high incidence of chromosome mis-segregation following treatment with nocodazole, and considering that we only scored one of 16 budding yeast chromosomes in this analysis, we propose that the reason why *bub1*Δ*K* cells are sensitive to antimicrotubule drugs, despite showing capacity to arrest in metaphase, is because of chromosome loss.

To more accurately quantitate their chromosome-loss rate, and to compare it with that of *sgo1Δ* mutants, we employed sectoring assays, which have previously been used to analyse many mitotic and checkpoint mutants [30,47]. We used a strain containing a nonessential test chromosome that carries the *SUP11* (ochre-suppressing tRNA) gene that makes colonies that are normally red, because of the *ade2–1* mutation, white. We scored loss of this chromosome at the first division by counting colonies that are half red and half white. Consistent with published data [30], in an unchallenged cell cycle, *bub1*Δ cells lost the test chromosome at a rate of 41 per 1,000 divisions compared to 0.5 per 1,000 in wild type. *bub1*Δ*K* cells also showed chromosome loss in a normal mitosis, but at a lower rate than *bub1*Δ, at 28 per 1,000 divisions (Figure 4E). The loss rate in *sgo1*Δ was very similar: 32 per 1,000 divisions.

If the Bub1 kinase domain and Sgo1p carry out the same function, one might predict little, if any, synthetic phenotype when combining the two mutations in *bub1*Δ*K*, *sgo1*Δ cells. Alternatively, if the double mutant was significantly sicker then that would suggest that the Bub1 kinase domain has other functions, in addition to Sgo1p targeting. Our analysis of the double mutant, both in terms of sensitivity to microtubule drugs and chromosome-loss rates, strongly supports the former option. We found that the double mutant is no more benomyl sensitive than the *bub1*Δ*K* strain (Figure 4D) and that the rate of chromosome loss was no higher than that of the *bub1*Δ*K* and *sgo1*Δ single mutants (Figure 4E).

These results show that the Bub1 kinase domain plays a role in chromosome segregation that becomes very important upon challenge with microtubule drugs. The lack of a synthetic genetic interaction suggests that the Bub1 kinase domain function is closely related to that of Sgo1p, consistent with the idea that the major role of the kinase is to efficiently target Sgo1p to centromeres (Figure 3). Note, *bub1*Δ cells have an even higher chromosome-loss rate because, in addition to their segregation defects, these cells have also lost their ability to checkpoint arrest.

### The Bub1 Kinase Domain Ensures Proper Chromosome Biorientation

**Figure 5:**
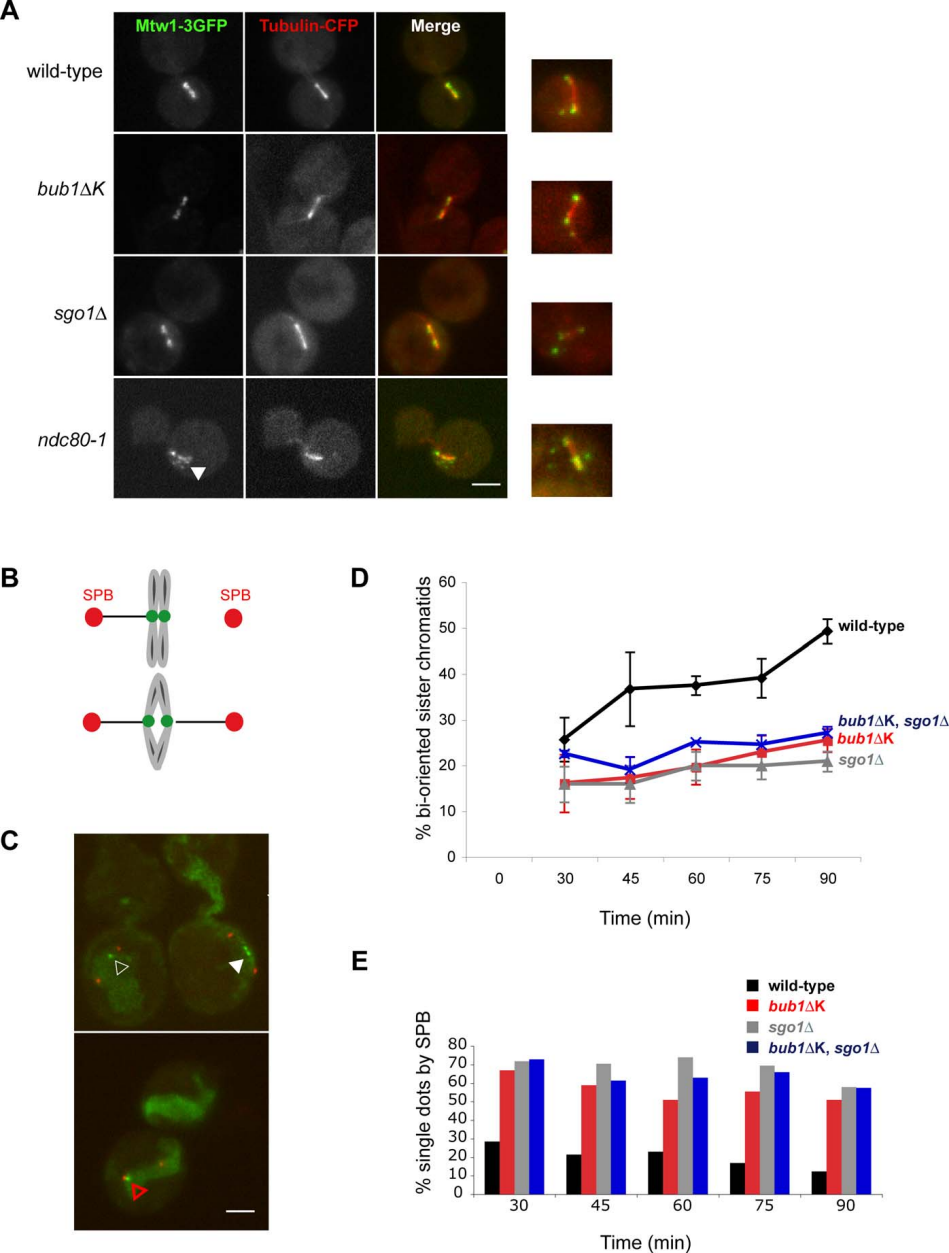
*bub1ΔK* and *sgo1Δ* Cells Show Defects in Establishment of Chromosome Biorientation (A) We analysed the indicated strains for unattached kinetochores following nocodazole treatment. Wild-type (SBY4340), *bub1*Δ*K* (JF123), *sgo1*Δ (JF202), and *ndc80–1* (SBY4342) cells were arrested in media containing 30 μg/ml benomyl and 15 μg/ml nocodazole for 3 h at 23 °C. The microtubule drugs were then washed out three times and released into rich media at 30 °C for 20 min. The control *ndc80–1* cells were grown at 36 °C for 1 h before fixation. Cells were then fixed in 3.7% formaldehyde for 5 min. We scored cells with kinetochores (Mtw1-3GFP staining) off the spindle axis as “unattached kinetochores.” All strains, apart from the *ndc80–1*, had mostly attached kinetochores aligned on the spindle. A total of 8% of wild-type cells contained unattached kinetochores at this timepoint (*n* = 212); *bub1*Δ*K*, 8% (*n* = 252); and *sgo1Δ*, 4% (*n* = 157) compared to *ndc80–1*, which had 79% (*n* = 96) unattached kinetochores. Right hand panel shows examples of unattached kinetochores for each strain. Scale bar represents 2 μm. (B) Schematic of the principle of the experiment showing GFP marked centromere “breathing” upon chromosome biorientation (i.e., two green dots). (C) Images of separated SPBs (Spc42-Tomato) containing either one GFP foci in between two SPBs (empty white triangle, scored as nonbioriented) or one GFP foci by one SPB (red empty triangle, scored as nonbioriented) or finally two GFP foci between two SPBs (filled triangle, scored as bioriented chromosome). Scale bar represents 2 μm. (D) Wild-type (JF152), *bub1*Δ*K* (JF154), *sgo1*Δ (JF156), and *bub1*Δ*K,sgo1*Δ (JF202) strains carrying cenIV-GFP, Met-Cdc20, and Spc42-Tomato were synchronised in G1 for 3 h and then depleted for Cdc20 by incubating them in α-factor and 8 mM methionine for 2 h before releasing them into media containing 30 μg/ml benomyl, 15 μg/ml nocodazole, and 8 mM methionine at 23 °C for 3 h. The nocodazole was then washed out and samples were taken at indicated times. Only cells that had separated SPBs were counted for each sample (*n* = 100) and scored for biorientated chromatids (two GFP foci between two SPBs) versus nonbioriented chromatids (one GFP foci between two SPBs and one GFP foci by one SPB). Error bars indicate standard deviation. Standard deviations are based on five separate experiments with all strains apart from *sgo1*Δ,*bub1*Δ*K* double mutant strain, which was used in two separate experiments. (E) This graph represents the cells in which the single GFP dot resided next to the SPB (red triangle) as opposed to in between the SPBs (white empty triangle), calculated as a percentage of the total cells with only one GFP dot. The data plotted are the average from two separate experiments.

As *bub1*Δ*K* cells have chromosome segregation defects, we tested whether they displayed kinetochore attachment defects. To do this we employed a *bub1*Δ*K* strain containing Tub1-cyan fluorescent protein (CFP) to label spindle microtubules and Mtw1-3xGFP to mark all kinetochores. Cells were arrested in mitosis with benomyl and nocodazole, which were then washed out, and the ensuing anaphase analysed. We identified no more unattached kinetochores (indicated by Mtw1-GFP foci off the spindle axis) in *bub1*Δ*K* cells than in wild-type cells (8%, see Figure 5A). Similar results, with low levels of unattached kinetochores, were obtained for *sgo1*Δ cells. *ndc80–1* mutants were used as positive controls for this experiment, and as expected these contain many unattached kinetochores at their restrictive temperature (Figure 5A [24,48]). From this we conclude that *bub1*Δ*K* and *sgo1*Δ cells do not display significant numbers of unattached kinetochores, even after spindle ablation and re-formation.

Next we wanted to test whether the *bub1*Δ*K* cells contain more subtle kinetochore attachment problems, such as defects in biorientation. If, during spindle reassembly, both sister kinetochores attach to microtubules from the same spindle pole (syntelically) perhaps the *bub1*Δ*K* cells would be unable to detect or correct this defect. Note syntelic attachments are unlikely to result in tension across sister kinetochores. To test this possibility, we used strains that carry an array of *tet* operators integrated only 2 kb from the centromere on Chromosome IV and express a Tet repressor-GFP fusion protein. In addition, their spindle poles are marked with Spc42-tomato, and the strain has a methionine repressible promoter for *CDC20* (p*MET*-*CDC20*). By depleting Cdc20p, we can induce a metaphase arrest that is independent of spindle damage and the checkpoint. Using such strains we could directly test the ability of cells to establish proper bipolar attachment after nocodazole treatment. Amphitelic attachments were visualised as pairs of GFP-centromere (CEN) spots pulled apart by the microtubule forces of the bipolar spindle (see Figure 5B) [49–51], whereas monotelic or syntelic attachments (both monopolar) remained as single GFP-CEN spots, on the spindle axis. Cells were arrested in G1, depleted of Cdc20p, and then released into media containing benomyl and nocodazole for 3 h. The microtubule drugs were then washed out, cells kept in metaphase by continued repression of Cdc20p, and the level of biorientation was scored as two GFP dots in between two red SPBs (filled white triangle, Figure 5C). This procedure gave us the relative levels of biorientation, although these numbers will be underestimated, since, at the time of fixation, some cells that have established biorientation will only show one GFP dot because of the “breathing” characteristics of mitotic centromeres [49,50]. Quantitation of these images revealed a significant defect in establishing proper biorientation in both *bub1*Δ*K* and *sgo1*Δ mutants, after nocodazole release, indicating that Bub1 kinase and Sgo1p both work to ensure efficient chromosome biorientation (Figure 5D). In addition, the *bub1*Δ*K,sgo1*Δ double mutant showed a very similar quantitative defect in biorientation to the two single mutants, suggesting that the Bub1 kinase domain and Sgo1p are necessary for the same biorientation function.

The position of the chromosomes in these images, relative to the SPBs (Figure 5E), is consistent with them being syntelically attached: both sisters are attached to the same spindle pole and therefore localise very close to it. We have carried out live-cell analysis of these cells (unpublished data) in which we scored the numbers of cells in which the GFP-marked chromosomes were “breathing” on a short mitotic spindle. Whilst 88% of chromosomes were observed to breathe in wild-type cells, only 56% did in the *bub1*Δ*K* and *sgo1*Δ mutants during the time observed. We conclude that these chromosomes are most likely syntelically attached, rather than being amphitelic attachments that fail to be stretched, and that the *bub1*Δ*K* and *sgo1*Δ mutants are unable to correct these attachments. Such a defect could account for much of the chromosome loss detailed in Figure 4. These findings agree well with recent live-cell imaging from the Murray lab showing that *sgo1–100* mutants frequently fail to correct syntelic attachments [52].

## Discussion

### Bub1 Kinase Is Not Required for Most Spindle Checkpoint Arrests, but Is Required for the Response to Reduced Cohesion

In this study we have carried out a detailed analysis of whether the kinase domain of Bub1 is required for a robust spindle checkpoint, using a truncated Bub1 kinase allele. Note, our truncation removes the last 413 residues of Bub1p (residues 609–1,021), which include 83 residues before the start of the conserved kinase domain. *bub1*Δ*K* cells showed sensitivity to benomyl, although not as severe as *bub1*Δ cells, and died rapidly in a nocodazole viability assay. However, our data clearly show that the Bub1 kinase domain is not required to initiate or maintain spindle checkpoint arrests induced by unattached or defective kinetochores. Robust arrests were observed in the presence of antimicrotubule drugs (either benomyl or nocodazole), and in the *mtw1–1* and *ndc80–1* kinetochore mutants (Figure 2A). In addition, we have found that the Bub1 kinase domain is unnecessary for the arrest induced by overexpression of the Mps1 protein kinase (*GAL-MPS1*, unpublished data and [53]). Thus, the Bub1 kinase domain is not necessary for a wide range of spindle checkpoint arrests.

However, like Sgo1p [39] and Ipl1p [23], the Bub1 kinase domain is necessary to delay anaphase onset in cells with reduced cohesion (Figure 2B). The simplest interpretation of this result is that these three functions are all needed for the checkpoint response to a lack of tension at kinetochores.

### The Sensitivity of *bub1*Δ*K* Cells to Microtubule Drugs Is Due to Chromosome Mis-Segregation after the Release from Nocodazole

In agreement with previous work [30] we found that *bub1*Δ*K* cells display chromosome mis-segregation in an unperturbed mitosis, at a level between those of wild-type and *bub1*Δ cells. The *bub1*Δ*K* chromosome-loss rate becomes far higher upon spindle damage (Figure 4). We have also demonstrated that *bub1*Δ*K* cells die rapidly when released from nocodazole. Importantly, this was not because of an inability to arrest in nocodazole, but rather to an inability to segregate chromosomes accurately upon spindle reassembly (see Figures 4 and 5). We propose that the Bub1 kinase domain has a role in regulating chromosome segregation every cell cycle, and that this role becomes particularly important after spindle damage. Such segregation defects could reflect the inability of *bub1*Δ*K* cells to respond to a lack of tension at kinetochores (Figure 2).

Our genetic studies support the idea that Bub1 kinase and Sgo1p act in the same pathway: whilst the complete gene deletions, *bub1*Δ and *sgo1*Δ are synthetically sick [35], we could detect no significant synthetic interaction between *bub1*Δ*K* and *sgo1*Δ in chromosome-loss rate (Figure 4E) sensitivity to antimicrotubule drugs (Figure 4D) or in the biorientation assay (Figure 5D). The simplest interpretation is that the important functions of Bub1 kinase are all Sgo1-dependent, and therefore already defective in the *sgo1*Δ. We propose that the complete gene double deletion (*bub1*Δ,*sgo1*Δ) is sicker because they are also unable to checkpoint arrest, which is supported by the finding that *mad2*Δ and *sgo1*Δ are also synthetically very sick [35].

### *Sgo1p* Is Mislocalised in *bub1*Δ*K* Cells

The close resemblance of the phenotypes between *bub1*Δ*K* and *sgo1*Δ cells encouraged us to investigate further whether they are part of the same pathway. Our data indicate that in budding yeast mitosis the kinase domain of Bub1p does play an important role in accurate Sgo1p localisation (Figure 3). Chromosome spreads and ChIP show that there were reduced levels of Sgo1 at centromeres, in strains lacking the Bub1 kinase domain.

In some experiments a slight increase in Sgo1p levels was observed on chromosome arms. If true for the chromosome as a whole, this finding would be consistent with observations in tissue culture where depletion of hsBub1 lead to hsSgo1 localizing to the chromosome arms [45]. In vertebrates this protects cohesion along the chromosome arms and perturbs sister-chromatid separation. However, in budding yeast there is no evidence that Sgo1 protects cohesin in mitotic cells, and when we analysed cohesin (Mcd1) cleavage in our *bub1*Δ*K* mutant (Figure S3) we saw no significant effect. General chromosome staining was not apparent in spreads of mitotic *bub1*Δ*K* cells, but we did frequently observe Sgo1p at spindle poles. The reason for this SPB localisation is currently unclear.

Overall, our data agree with work from other groups showing a role for Bub1 in localisation of Sgo1 to centromeres in mitosis in human cells [45,46], and in fission yeast [36]. Data are also in agreement with the finding that Bub1 kinase activity is required for centromeric localisation of Sgo1p (and the protein phosphatase PP2A sub-unit Rts1p) in budding yeast meiosis [44]. That study also showed that Bub1p localisation is independent of Sgo1p and PP2A [44].

### Is Sgo1 a Bub1 Kinase Substrate?

**Figure 6:**
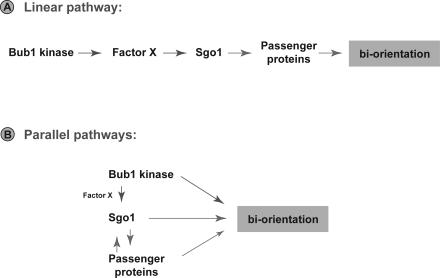
Models for Bub1 Kinase and Sgo1p Functions (A) Simple linear pathway: Bub1 kinase phosphorylates Factor X in such a way that Sgo1 is efficiently targeted to centromeres. This in turn ensures efficient recruitment of the Passenger proteins, which act to monitor and correct any inappropriate kinetochore-microtubule attachments. (B) Parallel pathways: Bub1 kinase, Sgo1p, and the Passenger proteins act in a concerted fashion to ensure efficient biorientation of sister kinetochores. This model better explains why localization of the different proteins are not entirely dependent upon one another. It also suggests that Bub1 kinase, and/or Sgo1p, might act to break inappropriate kinetochore attachments independently of Aurora kinase.

Because *sgo1*Δ and *bub1*Δ*K* cells have such similar phenotypes and Sgo1p is mislocalised in *bub1*Δ*K*, Sgo1p is a strong candidate to be a mitotic Bub1 kinase substrate. We have carried out preliminary experiments showing that Sgo1p is a phosphoprotein, but there is little if any effect on the phosphorylation state of Sgo1p in *bub1*Δ*K* mutants (J. Fernius, unpublished data). We also failed to observe a gel mobility change for fission yeast Sgo2p in *bub1* mutants (V. Vanoosthuyse, personal communication). Therefore we think it is likely that there is an unknown Bub1 kinase substrate (Factor X in our models, see Figure 6) that is required for Sgo1p targeting to centromeres.

Amongst the candidates for Factor X are the Chromosomal Passenger proteins (Aurora B, INCENP, and Survivin, see [54] for review). In *Drosophila* meiosis, *incenp* mutants perturb MEI-S332 (Sgo) localisation, and MEI-S332 was shown to be a good Aurora B substrate [55]. In budding yeast meiosis, Ipl1 kinase and the monopolin complex are key regulators of kinetochore orientation [56]. However, only partial perturbation of Sgo1p was observed upon Ipl1p depletion, and it was shown that Sgo1p actually recruits Ipl1p to meiotic centromeres [57]. Fission yeast studies also demonstrated an interdependence between Passenger proteins and Sgo2p targeting to centromeres, and in those experiments Survivin appears to be the Passenger protein most closely linked to Sgo2p [58,59].

Because of these links with the Passenger proteins, we have carried out preliminary experiments, looking at the Ipl1p and Sli15p in budding yeast mitotic cells that lack the Bub1 kinase domain. Whilst there may be subtle effects on the efficiency of recruitment of the Passenger proteins to mitotic centromeres, we find these to be far less significant than the effects on Sgo1p (Figure S4, and unpublished data). Thus we doubt that Factor X is one of the Passenger proteins (see models in Figure 6). Experiments to identify this factor are ongoing.

### Bub1 Kinase and Sgo1 Are Required for Efficient Chromosome Biorientation following Nocodazole Treatment

We have demonstrated that both *bub1*Δ*K* and *sgo1*Δ mutants have a defect in chromosome biorientation upon nocodazole release (Figure 5). In many of the mitotic cells we observed chromosomes that fail to biorient, lying close to one of the SPBs. One explanation could be that these mutants fail to detect or respond to inappropriate attachments, for example syntelic attachments that lack tension. This hypothesis is supported by the fact that *bub1*Δ*K* and *sgo1*Δ cells lack the ability to delay anaphase onset in response to tension defects induced by reduced cohesion (Figure 2 and [39]). Alternatively, *bub1*Δ*K* and *sgo1*Δ cells may sense these defects but be unable to break the inappropriate kinetochore-microtubule attachments. The Yen lab have recently demonstrated that human Sgo2 is not required to recruit Passenger proteins to centromeres but is needed to recruit the kinesin MCAK [60]. The activity of that kinesin, which is an Aurora B substrate, is known to be important for breaking inappropriate kinetochore attachments in some systems [61,62]. In addition, Sgo proteins have been shown to bind directly to microtubules and to modulate kinetochore-microtubule dynamics [63], so it is also possible that Sgo1p has a direct role in breaking microtubule attachments.

Bub1 kinase and Sgo1p could be part of the pathway that employs the Passenger proteins to destabilise kinetochore-microtubule attachments that lack tension. If so, one would expect similar phenotypes between *bub1*Δ*K*, *sgo1*Δ, and Passenger protein mutants. There are striking similarities: (1) *ipl1* and *sli15* mutants can arrest the cell cycle in response to unattached kinetochores because of nocodazole treatment, and (2) *ipl1* mutants fail to delay anaphase onset when cohesion is reduced and there is a lack of tension at kinetochores [23,64]. However, the Passenger proteins are essential, and are required for response to “core” kinetochore attachment defects, such as those present in *ndc80–1* and *mtw1–1* [24]. Figure 6 contains models that attempt to explain these observations.

In certain circumstances, such as those found during spindle assembly upon nocodoazole washout, the activity of Bub1 kinase and Sgo1p become necessary to ensure complete biorientation. Our data show that Bub1 kinase is required for efficient localisation of Sgo1p to centromeres, which in turn may aid efficient targeting of the Passenger proteins or their targets. Such a mode of action places Bub1p, Sgo1p and the Passengers in a simple linear pathway (Figure 6A). However, due to the lack of a clear effect on Passenger protein localisation, we currently favour an alternative model in which Bub1p, Sgo1p and the Passengers act cooperatively, but in distinct pathways, to ensure efficient biorientation (Figure 6B). The identification of direct Bub1 kinase substrates will be key to a deeper understanding of the role(s) of Bub1 kinase in the complex regulation of kinetochore attachment and error correction in mitosis.

## Materials and Methods

### Yeast strains, media, and standard techniques.

The yeast strains used in this study are derivatives of W303 (*ade2–1 his3–11 leu2–112 trp1–1 can1–100 ssdl-d2*) and are listed in Table 1. Yeast strains were grown in YPDA or selective media, and other basic yeast methods have been previously described. For synchronisation of cells in G1 we used 1 μg/ml α-factor for *bar1* and 10 μg/ml α-factor *BAR1* strains. Tagging of Spc42-Tomato (strains JF152, JF154, and JF156) was performed using cassettes described in [65,66].

**Table 1:**
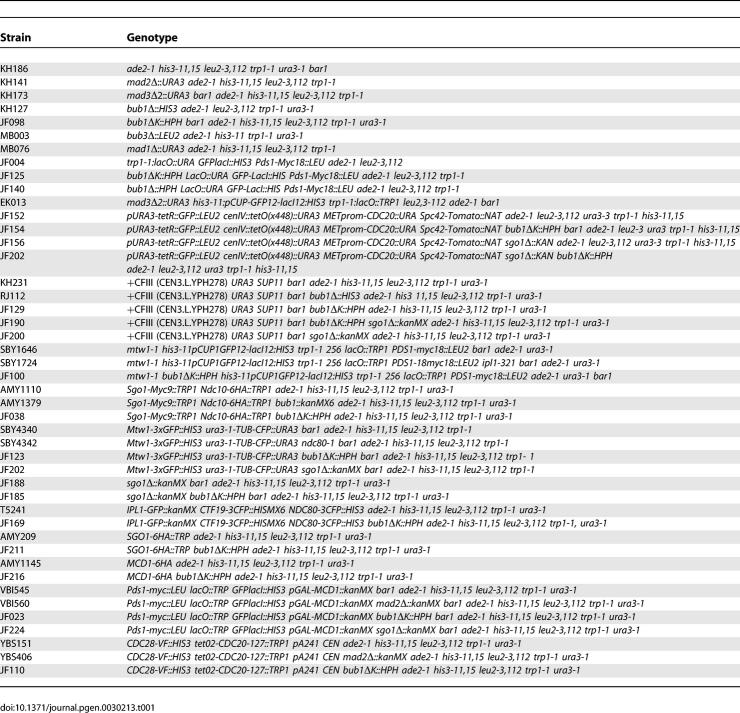
Yeast Strains

### Creation of *bub1ΔK* allele.

Amino acids 609–1,021 were truncated by PCR-mediated gene disruption, using pFA6a-Hygromycin resistance cassette [67] and verified by PCR and sequencing. The *sgo1Δ* strain was created using pFA6-kanMX6 [68].

### Checkpoint assays.

Benomyl, microcolony, and viability assays were all performed as previously described [9,41,69]. The *mtw1–1* checkpoint activation assay was performed as in [24]. The depletion of cohesin (lack of tension) assay was performed using *GAL-MCD1* as described [39].

A linear minichromosome assay was employed essentially as described in [39]. The linear chromosome used (pA241) is marked with *LEU2*. Such chromosomes have been shown to delay mitosis in a checkpoint-dependent manner [42] and were employed in a screen that identified *sgo1* alleles [39]. In the strains used here, and for the screen, the linear chromosome-induced delay becomes lethal as the cells also contain *CDC28-VF,* a mutation in *CDC28/CDK1* that reduces APC activity. This lethality is rescued by checkpoint mutations, as no mitotic delay is imposed. In addition, doxycycline represses a dominant *CDC20* allele that is insensitive to the spindle checkpoint proteins because it contains a mutation in the Mad2p binding site [13]. This means that all strains (wild-type or checkpoint mutant) containing the LMCs can be propagated in the absence of doxycycline. When doxycycline is added only wild-type *CDC20* is expressed: if the strain is checkpoint proficient it will not form colonies.

### Chromosome segregation assays.

Strains containing GFP-marked Chromosome IV was presynchronised in G1 and a sample was fixed in 3.7% formaldehyde for 5 min. The cells were then released into 30 μg/ml nocodazole and grown at 23 °C for 3 h. The nocodazole was then washed out, three times with YPDA media and then arrested in the next G1, and another sample was fixed as above. The same strain was used to analyse chromosome segregation at the first anaphase after nocodazole arrest. Cells were again presynchronised in G1 and arrested with nocodazole as above. Cells were released and fixed for 1 h in 3.7% formaldehyde and washed with 0.1 M potassium phosphate (pH 7.5). Then they were treated with 50 μg/ml Zymolyase 100,000 in 0.1 M potassium phosphate/0.7 M Sorbitol. General immunofluorescence was performed as previously described in [69]. Quantitative chromosome mis-segregation assay (sectoring assay) was performed as previously described [30,47].

### Chromosome spreads.

Chromosome spreads were performed on cycling cells and on nocodazole arrested cells as previously described by [70,71]. Sgo1-9myc was detected using rabbit anti-myc antibody (CM-100, Gramsch) at a 1:800 dilution, and anti-rabbit Alexa Fluor 488 (Invitrogen) at a 1:1,000 dilution. Ndc10-6HA was detected using a mouse anti-HA antibody (HA11, BabCO) at a 1:200 dilution and anti-mouse Alexa Fluor 594 (Invitrogen) at a 1:1,000 dilution. The spindle pole bodies were marked using an anti-Spc110 antibody at a 1:50 dilution (kind gift from John Kilmartin) and anti-mouse Alexa Fluor 594 (Invitrogen) at a 1:1,000 dilution.

### ChIP.

Cells were arrested for 3 h in 15 μg/ml nocodazole and 30 μg/ml benomyl, and 50 ml cells were collected for ChIP. *SGO1- 6HA* ChIPs were performed using 12CA5 anti-HA antibody (Roche), and the general protocol including primers was performed as described in [72].

### Chromosome attachment assay.

Similar experiments to those described in [24] were performed, where the mitotic spindle is labelled with tubulin-CFP and the kinetochores with Mtw1-3xGFP. Strains were treated with 15 μg/ml nocodazole and 30 μg/ml benomyl at 23 °C for 3 h. Cells were then washed three times and grown in YPDA at 30 °C for 20 min, then fixed in 3.7% formaldehyde for 5 min. *ndc80–1* cells were grown for the last 90 min at the restrictive temperature of 36 °C.

### Chromosome biorientation assay.

Strains were arrested in G1 in medium lacking methionine for 3 h, then transferred to YPDA media plus 8 mM methionine for 2 h at 30 °C to deplete Cdc20. The α-factor was subsequently washed out, and cells were then incubated at 23 °C for 3 h in media containing 30 μg/ml benomyl, 30 μg/ml nocodazole, and 8mM methionine. The microtubule drugs were then washed out, and the spindle was allowed to reform at 30 °C in YPDA media plus 8 mM methionine. Cells were fixed at indicated times in 3.7% formaldehyde for 5 min. The GFP dots were analysed only in cells that had a short bipolar spindle (i.e., two SPBs) to score for biorientation. These experiments were repeated at least three times, and at each time point 100 cells were counted.

## Supporting Information

Figure S1 *bub1*Δ*K* Cells Initiate and Maintain a Robust Checkpoint Arrest in Response to Antimicrotubule Drugs (A) Wild-type (KH186), *bub1*Δ*K* (JF098), *bub1*Δ (KH127), and *mad2*Δ (KH141) yeast strains were analysed for growth on YPDA media containing (A) 20 μg/ml or (B) 80 μg/ml benomyl grown at 23 °C. We scored the percentage of cells that remain arrested (large-budded) throughout the time course (*n* = 50 cells). The *bub1*Δ*K* cells initiate and maintain a robust checkpoint arrest.
(49 KB PDF)

Figure S2 Sgo1 Localisation to Kinetochores (Ndc10) Is Defective in *bub1*Δ*K*
(A) Wild-type (AMY1110), *bub1*Δ*K* (JF038), and *bub1*Δ (AMY1379) cells were harvested in log-phase, and staining was performed on chromosome spreads. Anti-myc (CM-100) was used to detect Sgo1-9myc, and anti-HA (HA11) was used to detect Ndc10-6HA. Cells with clear Ndc10-6HA staining were categorised as either having colocalisation with Sgo1-9myc (left panel), or only partial colocalisation that could be due to spindle pole bodies (right panel). The spreads with no Sgo1 staining are not shown but were similar in number in wild type and *bub1*Δ*K* mutant.
(B) Quantification of spreads scoring percentage of spreads that showed colocalisation of Sgo1 to the Ndc10 kinetochore marker. Error bars indicate standard deviation of the mean.
(66 KB PDF)

Figure S3 Mcd1 Cleavage Is Not Defective in *bub1*Δ*K* Cells Wild-type (AMY1145) and *bub1*ΔK (JF216) strains were arrested in mitosis using 15 μg/ml nocodazole and 30 μg/ml benomyl at 23 °C for 3 h. The drug was washed out and samples for immunoblotting with 12CA5 (anti-HA antibody) were taken at indicated timepoints. There was no significant difference in timing or amount of Mcd1 cleavage detected. Blot shows a representative experiment. The experiment was repeated three times.
(124 KB PDF)

Figure S4 Kinetochore Localisation of Ipl1 Kinase Is Not Significantly Perturbed in *bub1*Δ*K* Cells Wild-type (T5241) and *bub1*Δ*K* (JF169) strains were arrested in mitosis using 15 μg/ml nocodazole and 30 μg/ml benomyl. Kinetochores were marked using Ctf19-CFP and Ndc80-CFP. There was no significant reduction of Ipl1-GFP on kinetochores in the *bub1*Δ*K* strain compared to wild-type cells.
(196 KB PDF)

## Abbreviations

APC/C: Anaphase Promoting Complex or Cyclosome
ChIP: chromatin immunoprecipitation
CFP: cyan fluorescent protein
GFP: green fluorescent protein
SPB: spindle pole body
