# Supplementary material for: Bub1 Kinase Targets Sgo1 to Ensure Efficient Chromosome Biorientation in Budding Yeast Mitosis
Source: PLoS Genet. 2007 Nov 30;3(11):e213. doi: 10.1371/journal.pgen.0030213 (PMC2098806; doi:10.1371/journal.pgen.0030213)
Supplement: Figure S1 — (A) Wild-type (KH186), bub1ΔK (JF098), bub1Δ (KH127), and mad2Δ (KH141) yeast strains were analysed for growth on YPDA media containing (A) 20 μg/ml or (B) 80 μg/ml benomyl grown at 23 °C. We scored the percentage of cells that remain arrested (large-budded) throughout the time course (n = 50 cells). The bub1ΔK cells initiate and maintain a robust checkpoint arrest. (49 KB PDF) [file pgen.0030213.sg001.pdf]

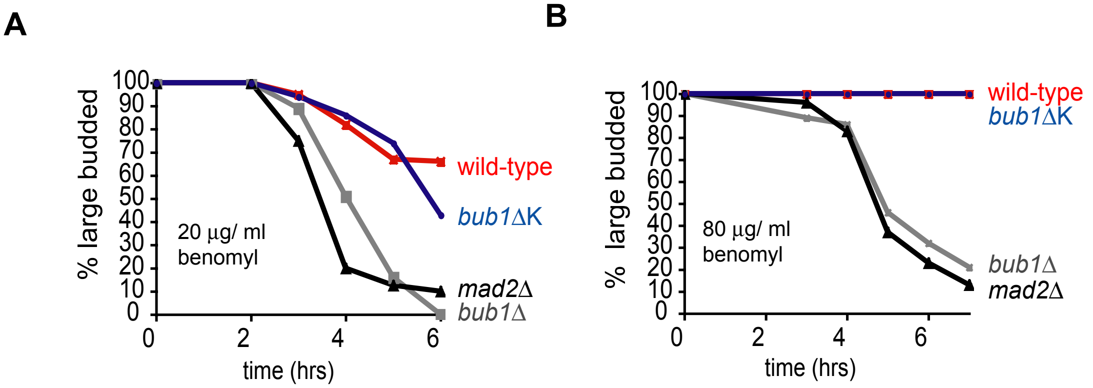

Figure S1. (A) Wild-type (KH186), *bub1ΔK* (JF098), *bub1Δ* (KH127) and *mad2Δ* (KH141) yeast strins were analysed for growth on YPDA media containing (A) 20 µg/ml or (B) 80 µg/ml benomyl grown at 23°C. We scored the percentage of cells that remain arrested (large-budded) throughout the time course (n=50 cells). The *bub1ΔK* cells initiate and maintain a robust checkpoint arrest.
