# Supplementary material for: Bub1 Kinase Targets Sgo1 to Ensure Efficient Chromosome Biorientation in Budding Yeast Mitosis
Source: PLoS Genet. 2007 Nov 30;3(11):e213. doi: 10.1371/journal.pgen.0030213 (PMC2098806; doi:10.1371/journal.pgen.0030213)
Supplement: Figure S2 — (A) Wild-type (AMY1110), bub1ΔK (JF038), and bub1Δ (AMY1379) cells were harvested in log-phase, and staining was performed on chromosome spreads. Anti-myc (CM-100) was used to detect Sgo1-9myc, and anti-HA (HA11) was used to detect Ndc10-6HA. Cells with clear Ndc10-6HA staining were categorised as either having colocalisation with Sgo1-9myc (left panel), or only partial colocalisation that could be due to spindle pole bodies (right panel). The spreads with no Sgo1 staining are not shown but were similar in number in wild type and bub1ΔK mutant. (B) Quantification of spreads scoring percentage of spreads that showed colocalisation of Sgo1 to the Ndc10 kinetochore marker. Error bars indicate standard deviation of the mean. (66 KB PDF) [file pgen.0030213.sg002.pdf]

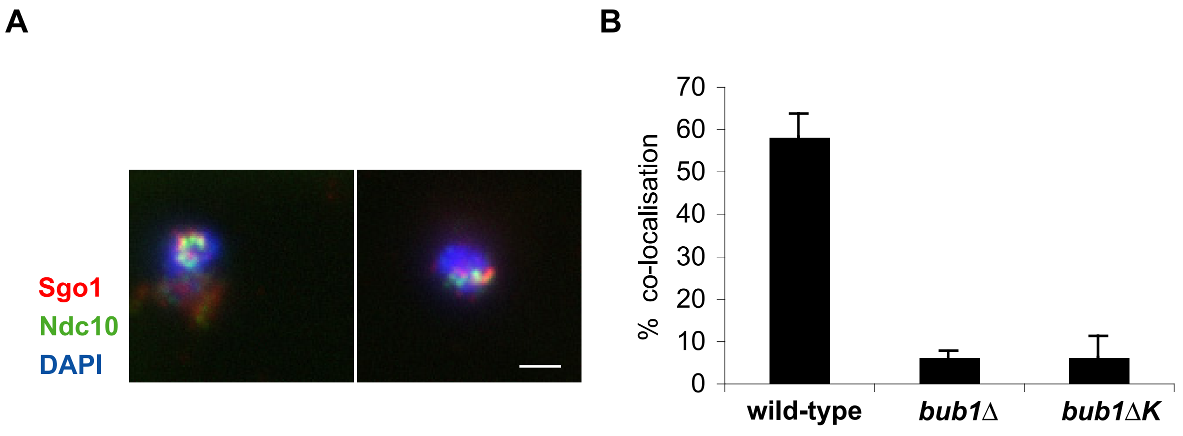

Figure S2. Sgo1 localisation to kinetochores (Ndc10) is defective in *bub1ΔK*. (A) Wild-type (AMY1110), *bub1ΔK* (JF038) and *bub1Δ* (AMY1379) cells were harvested in log-phase and staining was performed on chromosome spreads. Anti-myc (CM-100) was used to detect Sgo1-9myc and anti-HA (HA11) was used to detect Ndc10-6HA. Cells with clear Ndc10-6HA staining were categorised as either having co-localisation with Sgo1-9myc (left panel), or only partial co-localisation that could be due to spindle pole bodies (right panel). The spreads with no Sgo1 staining are not shown but were similar in number in wild-type and *bub1ΔK* mutant. (B) Quantification of spreads scoring percentage of spreads that showed co-localisation of Sgo1 to the Ndc10 kinetochore marker. Error bars indicate standard deviation of the mean.
