# Supplementary material for: Bub1 Kinase Targets Sgo1 to Ensure Efficient Chromosome Biorientation in Budding Yeast Mitosis
Source: PLoS Genet. 2007 Nov 30;3(11):e213. doi: 10.1371/journal.pgen.0030213 (PMC2098806; doi:10.1371/journal.pgen.0030213)
Supplement: Figure S3 — Wild-type (AMY1145) and bub1ΔK (JF216) strains were arrested in mitosis using 15 μg/ml nocodazole and 30 μg/ml benomyl at 23 °C for 3 h. The drug was washed out and samples for immunoblotting with 12CA5 (anti-HA antibody) were taken at indicated timepoints. There was no significant difference in timing or amount of Mcd1 cleavage detected. Blot shows a representative experiment. The experiment was repeated three times. (124 KB PDF) [file pgen.0030213.sg003.pdf]

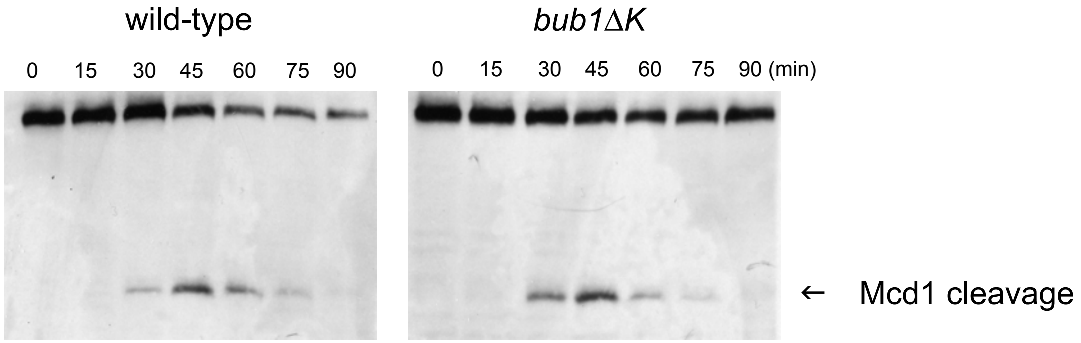

Figure S3. Mcd1 cleavage is not defective in *bub1ΔK* cells. Wild-type (AMY1145) and *bub1Δ*K (JF216) strains were arrested in mitosis using 15 μg/ml nocodazole and 30 μg/ml benomyl at 23ºC for 3 hours. The drug was washed out and samples for immunoblotting with 12CA5 (anti-HA antibody) were taken at indicated timepoints. There was no significant difference in timing or amount of Mcd1 cleavage detected. Blot shows a representative experiment. The experiment was repeated 3 times.
