# Supplementary material for: Bub1 Kinase Targets Sgo1 to Ensure Efficient Chromosome Biorientation in Budding Yeast Mitosis
Source: PLoS Genet. 2007 Nov 30;3(11):e213. doi: 10.1371/journal.pgen.0030213 (PMC2098806; doi:10.1371/journal.pgen.0030213)
Supplement: Figure S4 — Wild-type (T5241) and bub1ΔK (JF169) strains were arrested in mitosis using 15 μg/ml nocodazole and 30 μg/ml benomyl. Kinetochores were marked using Ctf19-CFP and Ndc80-CFP. There was no significant reduction of Ipl1-GFP on kinetochores in the bub1ΔK strain compared to wild-type cells. (196 KB PDF) [file pgen.0030213.sg004.pdf]

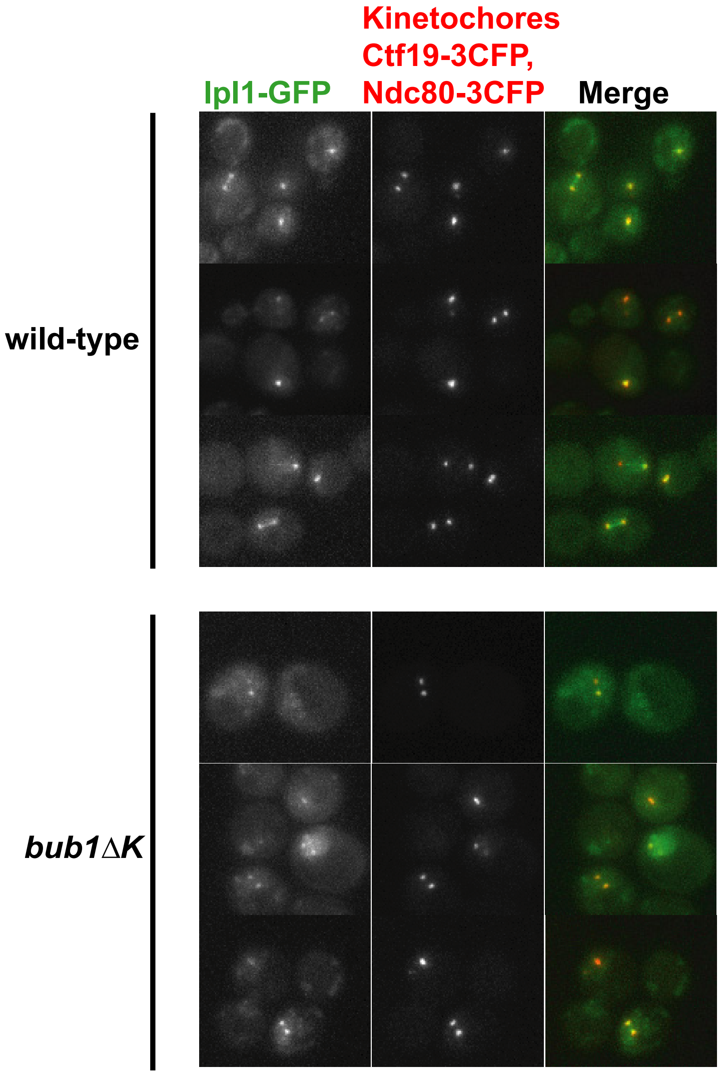

Figure S4. Wild-type (T5241) and *bub1ΔK* (JF169) strains were arrested in mitosis using 15 µg/ml nocodazole and 30 µg/ml benomyl. Kinetochores were marked using Ctf19-CFP and Ndc80-CFP. There was no significant reduction of Ipl1-GFP on kinetochores in the *bub1ΔK* strain compared to wild-type cells.
